# Pathological findings in spontaneously dead and euthanized sows – a descriptive study

**DOI:** 10.1186/s40813-019-0132-y

**Published:** 2019-11-20

**Authors:** Eve Ala-Kurikka, Camilla Munsterhjelm, Paula Bergman, Taina Laine, Henna Pekkarinen, Olli Peltoniemi, Anna Valros, Mari Heinonen

**Affiliations:** 1 Research Centre for Animal Welfare, Department of Production Animal Medicine, Faculty of Veterinary Medicine, University of Helsinki, P.O. Box 57, 00014 Helsinki, Finland; 2 Department of Production Animal Medicine, Faculty of Veterinary Medicine, University of Helsinki, Paroninkuja 20, Saarentaus, 04920 Mäntsälä, Finland; 3 Veterinary Bacteriology and Pathology Unit, Finnish Food Authority (until 31 December 2018 Finnish Food Safety Authority Evira), Mustialankatu 3, 00790 Helsinki, Finland; 4 Department of Veterinary Biosciences, Faculty of Veterinary Medicine, University of Helsinki, P.O. Box 66, 00014 Helsinki, Finland

**Keywords:** Sow, Mortality, On-farm death, Euthanasia, Post-mortem examination, Lameness, Locomotor disorder, Degenerative joint disease, Dental and periodontal disease

## Abstract

**Background:**

A high rate of euthanized and spontaneously dead sows causes production losses and likely indicates underlying welfare problems. Identification of predisposing factors to on-farm deaths requires a thorough understanding of the causes. Post-mortem examination is needed for a proper diagnosis. The aims of this descriptive study were to determine causes of spontaneous deaths and euthanasia in sows in a convenience sample of Finnish herds and to describe pathological findings in the locomotor system and in teeth and gums.

**Results:**

This study described post-mortem findings in 65 sows found dead or euthanized on 15 farms. All but one of the sows presented with two or more pathological findings. The majority of primary pathologic-anatomic diagnoses (PAD-1) were inflammatory. The most prevalent diagnoses were arthritis and peritonitis (9% of sows each). The locomotor system was the body part most commonly affected by lesions. Findings in the locomotor system unassociated with death were present in 85% of the animals, additionally 29% of PAD-1 s concerned the locomotor system. The prevalence for both degenerative joint disease and tooth wear was 71%. Farmers had noted clinical signs within 30 days of death in every euthanized sow and in half of the spontaneously dead ones. The farmer’s impression of the cause of death agreed at least partly with the PAD-1 in 44% of the cases.

**Conclusion:**

Multiple pathologies were the norm in the present animals. This may indicate an extended course of illness and therefore also an unnecessary delay in medical treatment or euthanasia. The prevalence and clinical relevance of the most common disorders, including degenerative joint disease and tooth wear, need to be elucidated.

## Background

The replacement rate for sows in commercial production is generally high. A Swedish study including 21 commercial herds replaced roughly half of their sows annually [1]. A high rate of involuntary cullings and mortality of sows causes production losses and likely indicates underlying welfare problems on the farm. The desired type of removal is planned, usually due to old age or low productivity [2, 3]. Unplanned removals include on-farm deaths (OFDs), that is animals euthanized or found dead. Sow OFD is included as an animal-based farm-level welfare indicator in the Welfare Quality® assessment system [4]. According to a Belgian study, consumers also identify mortality as an indicator of welfare [5].

It is difficult to compare OFD levels from different sources. There is no established methodology, and scientific reports often lack essential details on methods. Different brands of production software calculate OFD figures in different ways. Reported OFD levels in Danish data sets have been in the range of 3.9–15.6% [6, 7]. Sow mortality in Finnish pig herds was 6.7% in 2016 according to the national swine herd health register Sikava, which covers 90% of herds in the country [8]. In a data set of 126 Finnish farms visited for assessment of sow welfare according to Welfare Quality® between 2011 and 2017, upper and lower quartiles of spontaneous deaths in sows were 4.1 and 1.0%, respectively (Munsterhjelm C., unpublished data).

Literature on causes of OFDs in sows is scarce, hindering their comparison, as is the case for OFD rates. Retrospective analyses of field data appear to suffer from a number of shortcomings regarding data recording. Post-mortem examinations (PMEs) are, for example, undertaken according to different protocols, which often are not described in sufficient detail. A Danish study reported lesions in the gastrointestinal system, spleen and reproductive system as the main causes for spontaneous death [9]. Danish and Swedish data, the latter from one farm only, showed that disorders in the locomotor system were the most frequent reason for euthanizing sows [9, 10].

Decreasing sow OFD will affect both farm economy and animal welfare positively. Identification of predisposing factors requires a thorough understanding of the causes. Data available on farms tend, however, to be of poor or at least variable quality. Notes on circumstances surrounding cases of OFD are seldom collected in a standardized way, and the ability of the stockperson to interpret signs of diseases varies. Thomsen et al. [11] compared farmers´ perceptions of the cause of death with necropsy results in dairy cows and concluded that farmers were correct in about 50% of cases. Thus, we believe that the causes for sow OFD are poorly understood by both farmers and veterinarians on most farms.

This research was designed to gain insight into the causes for sow OFD in a sample of Finnish farms. We performed PME in order to establish a proper diagnosis. PME may clarify the sequence of events even if the proximate cause of death is known [11], and its diagnostic value exceeds that of other types of samples submitted to laboratories [12].

The main aim of this descriptive study was to determine causes of OFDs in sows in a convenience sample of Finnish herds. A second aim was to describe pathological findings with a special emphasis on the locomotor system and dental and periodontal disease (DPD). These areas of emphasis were selected based on a high prevalence of findings in the study animals. The lack of scientific literature on DPD served as the rationale for reporting the current findings. The third aim was to analyse to what degree farmers’ perceptions of the causes of OFD agree with pathological-anatomical diagnoses.

## Methods

### Animals and herds

This research is part of a sow longevity study collecting data from client herds of the three largest slaughterhouses in Finland during 2014–2016. The reference population thus included the majority of Finnish pig production. The study population was formed by voluntary herds, the owners of which were willing to provide the research group with sow carcasses and the necessary anamnestic information. Recruitment proved difficult as herd owners were uncertain regarding the extra work involved. The recruitment process included active contacting of farms by different operators in the business, including the research team and the national producers’ organization (for details see Heinonen et al. [13]).

The final data set was collected from 15 herds from the southern half of Finland and included 65 sows, 38 of which were euthanized and 27 found dead. Most herd owners provided two sows (median 2, range 1–20 sows per herd). Sow characteristics are given in Table 1. Although the researchers communicated a wish to examine all sows dead on the farm over a certain time period, the herd owners were free to decide which sows to enrol in the study. They informed the research personnel about death or imminent euthanasia of a sow by e-mail or telephone. The sow was included in the study if a necropsy could be arranged on the day following death at the latest.

**Table 1:** Effects of the means of death (found dead vs. euthanized) on characteristics of the animals and pre-mortem circumstances in 65 sows found dead or euthanized on 15 Finnish farms

	Found dead, *n* = 27	Euthanized, *n* = 38	Test and significance
Parity, median (min–max)	2 (0–9)	3 (0–10)	*p* > 0.1, T-test
Age, median (min-max)	621 days (330–1823)	643 days (340–1938)	*p* > 0.1, T-test
Body condition score, average (std. dev.)	2.9 (0.79)	2.5 (0.92)	*p* = 0.047 T-test
Weight, average (std. dev.)	244 kg (52.8, *n* = 24)	219 kg (52.4, *n* = 28)	*p* > 0.1, T-test
Last event before death, n (%^a^)			
Insemination	19 (70%)	13 (34%)	
Farrowing	6 (22%)	15 (39%)	
Weaning	2 (7%)	10 (26%)	
Last place of residence, n (%^a^)			
Group housing	15 (56%)	17 (45%)	
Gestation stall^b^	2 (7%)	1 (3%)	
Farrowing pen	10 (37%)	20 (53%)	
Clinical signs < 30 days before death, n (%^a^)			
Yes	14 (52%)	38 (100%)	*p* < 0.001, x^2^-test
No	13 (48%)	0	
Medically treated for sickness < 30 days before death, n (%^a^)			
Yes	10 (37%)	24 (63%)	*p* = 0.04, x^2^-test
No	17 (63%)	14 (37%)	

^a^Proportion of sows within means of death, ^b^Stall housing is by law allowed only from weaning to 4weeks of gestation. x^2^ is the Chi-squared test

A questionnaire (provided as a Additional file 1) was devised to collect general information on the sow and on signs and circumstances preceding death, including the stockperson’s perception of the cause of death. The questions were selected based on literature and clinical experience of the research group. Qualitative questions were mostly closed, but free text was allowed. The majority of the information was collected by telephone or personal interview by one researcher, but in a few cases the questionnaire was completed by the farmer and mailed.

### Post-mortem examination and classification of findings

The place of PME was determined according to the location of the farm as well as practical constraints. PMEs were performed at the Finnish Food Safety Authority in Seinäjoki (*n* = 23), at the University of Helsinki (*n* = 37), at the University Ambulatory Clinic in Mäntsälä (*n* = 3) or on the farm (*n* = 2). The animal was weighed or for animals necropsied in the ambulatory clinic or on-farm the body weight was estimated. Body condition score (BCS) was evaluated using a 5-grade scale (1 = cachectic; 3 = normal; 5 = obese).

PMEs were performed by 14 veterinarians. At the Finnish Food Safety Authority and University of Helsinki, full necropsy was performed, with the examination of the vertebral column (by sawing in half) if no clear reason for death/signs was found in other organs. In the ambulatory clinic and on-farm, all internal organs were examined macroscopically. Special emphasis was placed to teeth, stomach and joints, and all animals were thoroughly examined regarding these sites. All teeth were examined by opening the cheeks. The stomach was fully opened, delicately rinsed and examined for ulcerations. Samples for histology were taken from heart, lung, liver, kidneys, urinary bladder, uterus and affected internal organs, fixed in 10% neutral buffered formalin, embedded in paraffin wax and sectioned (4 μm) for staining (haematoxylin and eosin). The standard operating procedure (SOP) for PME is provided as a Additional file 2.

In general, lesions were considered inflammatory when macroscopic changes including purulent exudate or severe hyperemia were evident, or the finding could be confirmed by histopathological examination. The cause of death was categorized “cardiogenic”, if macroscopical or histological lesions in the heart were noted. The cause of death was characterized as “unknown, suspected cardiogenic” in cases where: 1) the animal had died naturally, 2) acute pulmonary edema was noted, 3) moderate to severe passive congestion of lungs and liver was noted both macroscopically and microscopically, and 4) no other macroscopic or microscopic cause for death or agony was seen. Decubital ulcers (DUs) were macroscopically classified according to a 4-grade scale according to Jensen [14]: the ulceration of skin was (1) limited to epidermis; (2) included dermis; (3) included subcutaneous tissue; and (4) exposed the bone. Since an accurate differentiation between epidermal and dermal ulceration is impossible with macroscopical examination alone, the ulcers were pooled into two groups, one group including grades 1 to 2 and the second group including grades 3 to 4. Lesions in the *Pars esophagea* of the stomach were recorded according to a scale described by Hautala and Rautiainen [15].

Abnormalities in shoulder (humeral), elbow (humeroulnar and humeroradial), hip (coxofemoral) and knee (femorotibial and femoropatellar) joints were described in detail, including the amount (increased/ not increased) and appearance of synovial fluid (clear/ cloudy/ reddish/ purulent), the appearance of the synovial membrane (reddish/ proliferated/ folded) and the joint surfaces (uneven/ eroded/ craters/ detached pieces of joint cartilage/ changes indicating trauma). Cloudy or purulent synovial fluid and changes of synovial membrane indicative of inflammation were regarded as inflammatory arthritis. The presence of gross lesions (at least erosion or thinning of the joint cartilage) in one or more joint surfaces without changes indicating an acute inflammation of the joint was regarded as degenerative joint disease. Mild cases with only colour changes, mild thickening of synovial membranes or an increased amount of joint fluid were regarded as other joint disease.

Pathological findings in the teeth and gums were recorded on a dental chart. Dental and periodontal disease (DPD) comprised four variables, including tooth wear, dental calculus, tooth fracture and periodontal disease. Periodontal disease was assessed macroscopically and included gingivitis, periodontitis and/or at least one loose or missing tooth. A given condition was considered present if at least one tooth was affected to a degree that was evaluated as at least moderate.

For each sow, pathological findings were classified according to their assumed role in the process leading to death or euthanasia. The main cause of death was considered the primary pathological-anatomical diagnosis (PAD-1). In cases with evidence of more than one contributing cause, PAD-1 was the event assumed to have occurred latest. For example, if a gastric ulcer had progressed to peritonitis, PAD-1 was peritonitis. Gastric ulcer in this example would have been the secondary cause of death or PAD-2. PAD-2 was defined as a secondary pathological-anatomical diagnosis, assumed to be less important than or preceding PAD-1. Other PME findings, which logically could have contributed to the health status of the sow but were not directly related to death, were considered incidental findings.

The farmer-reported cause of death was classified as correct if it completely agreed with the PAD-1. A perception was considered partly correct if, for example, the farmer reported paralysis and the PAD-1 was osteomyelitis in the vertebral column.

### Statistical analysis

Statistical analyses were performed using SPSS software (IBM SPSS Statistics for Macintosh, version 21.0 and for Windows, version 24.0; IBM Corp., Armonk, NY, USA).

Differences in animal characteristics, frequency of clinical signs and medical treatments between spontaneously dead and euthanized sows were analysed. For the continuous and normally distributed variables of parity, age, BCS and weight, the T-test was applied. Differences in frequencies of animals with clinical signs and medically treated animals were analysed using the Chi-squared test.

## Results

### Characteristics of the animals and pre-mortem circumstances

Sow and pre-mortem data are summarized in Table 1. The median sow included in this study was 643 days old (range 330–1938) and had a parity of 3 (0–10), weighed 230 kg (143–370) and had a BCS of 3 (1–5). Of these characteristics, only BCS differed between the means of death, with euthanized sows being thinner than spontaneously dead ones (average 2.5 vs. 2.9; *p* = 0.047, T-test).

Spontaneous deaths were distributed within the three production phases (gestation, lactation, weaning to insemination) according to their relative length. There was, however, a hint of clustering with 4 of altogether 19 deaths during gestation in the very latest phase, when the sow was already moved to the farrowing pen. Cases of euthanasia appeared heavily clustered to the days between weaning and insemination, with 26% of cases (*n* = 10) taking place during a period that usually comprises only 1 week of the 21– to 22–week production cycle.

**Fig. 1:**
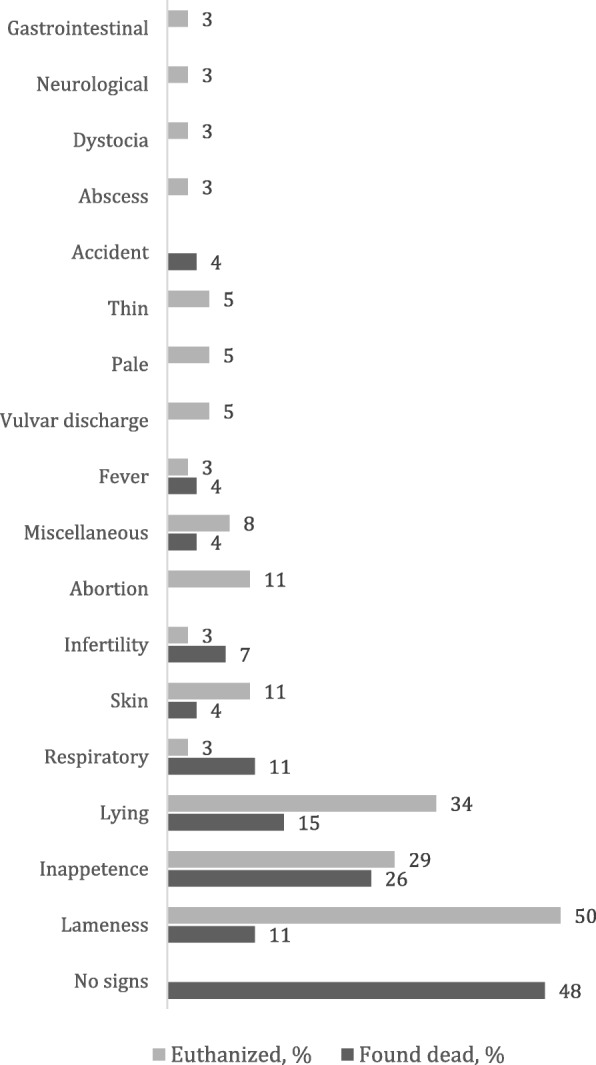
Frequencies of clinical signs in sows within 30 days preceding death (*n* = 27) or euthanasia (*n* = 38) observed by the farmer on 15 Finnish farms. More than one sign per sow could be reported

Clinical signs observed by the farmer within 30 days preceding death were more common in euthanized sows than in those found dead (100% vs. 52%; *p* < 0.001, x^2^ (1)=22.87). The most commonly reported signs included lameness (*n* = 21), inappetence (*n* = 17) and inability to stand up without assistance (*n* = 17; Fig. 1). The only sign that appeared to predict the category of PAD-1 well was lameness, which was reported in 16 of 19 animals with a PAD-1 affecting the locomotor system (Table 2). The farmer-reported lame leg and the leg with the worst pathological condition matched in 17 (57%) of 30 sows that were lame or unable to stand up without assistance. If a PAD affected both hind legs, mention of either of them was considered a match. All farmer-reported signs are given in Fig. 1, and the most common signs per PAD-1 are presented in Table 2.

**Table 2:** Most common clinical signs observed by the farmer in sows (*n* = 65) within 30 days preceding death or euthanasia, according to primary pathological-anatomical diagnosis (PAD-1) category. Prevalences are given as number of sows with the finding (proportion within PAD-1 category). More than one sign per sow could be reported

PAD-1 category	Lameness	Lying^a^	Inappe-tence	Fever	Uterine discharge	Respira-tory	No signs
Inflammatory, other than locomotor (*n* = 20)	0	2 (10%)	6 (30%)	2 (10%)	2 (10%)	1 (5%)	7 (35%)
Locomotor, inflammatory (*n* = 11)	9 (82%)	5 (46%)	4 (36%)	0	0	0	0
Locomotor, non-inflammatory (*n* = 8)	7 (88%)	3 (38%)	2 (25%)	0	0	0	1 (13%)
Cardiogenic (*n* = 3)	1 (33%)	1 (33%)	2 (66%)	0	0	0	0
Miscellaneous (*n* = 8)	1 (13%)	0	2 (25%)	0	0	0	2 (25%)
Unknown, susp. cardiogenic	1 (17%)	1 (17%)	1 (17%)	0	0	2 (33%)	1 (17%)
Unknown (*n* = 9)	3 (33%)	5 (56%)	1 (11%)	0	0	1 (11%)	2 (22%)

^a^Unable to stand up without assistance

Medical treatment within 30 days preceding death was significantly more common in animals euthanized than in those found dead (63% vs. 37%; *p* = 0.04, x^2^ (1)=4.32).

### Post-mortem findings and farmers’ perceptions of the causes of death

PAD-1 and PAD-2 are given in Table 3. The most common PAD-1 s were arthritis and peritonitis, which were present in 6 sows (9%). No PAD could be determined in 11 sows (17%). Looking at categorized PAD-1, roughly half (*n* = 31) of the animals presented with an inflammatory disorder, one-third of which was located in the locomotor system (*n* = 11), followed by ‘locomotor, non-inflammatory’ in 8 sows (12%), ‘miscellaneous’ in 8 sows (12%). In six sows (9%), the cause of death was categorized “unknown, suspected cardiogenic”.

**Table 3:** Primary and secondary pathological-anatomical diagnoses (PAD-1 and PAD-2) and PAD categories in post-mortem examination of 65 sows found dead (*n* = 27) or euthanized (*n* = 38) from 15 Finnish sow herds

PAD category and diagnosis	PAD-1			PAD-2		
	% of all sows	Found dead, n	Eutha-nized, n	% of all sows	Found dead, n	Eutha-nized, n
**Inflammatory, other than locomotor**	**30.8**	**11**	**9**	**9.2**	**3**	**3**
Peritonitis	9.2	5	1	0	0	0
Abscess	4.6	0	3	0	0	0
Pneumonia, pleuritis or bronchopneumonia	4.6	3	0	3.1	1	1
Generalized infection	3.1	1	1	0	0	0
Metritis, endometritis or pyometra	3.1	0	2	1.5	1	0
Cystitis, chronic	1.5	0	1	1.5	0	1
Enteritis and haemorrhagic enteritis	1.5	1	0	1.5	0	1
Pericarditis (with or without pneumonia)	1.5	0	1	1.5	1	0
Septic shock	1.5	1	0	0	0	0
**Locomotor, inflammatory**	**16.9**	**0**	**11**	**1.5**	**0**	**1**
Arthritis	9.2	0	6	0	0	0
Osteomyelitis, vertebral column	3.1	0	2	0	0	0
Myositis or cellulitis	3.1	0	2	0	0	0
Digital dermatitis	1.5	0	1	0	0	0
Periarthritis	0	0	0	1.5	0	1
**Locomotor, non-inflammatory**	**12.3**	**1**	**7**	**7.7**	**0**	**5**
Fracture	7.7	1	4	1.5	0	1
Callus in the hoof	1.5	0	1	0	0	0
Arthrosis	1.5	0	1	6.2	0	4
Myositis due to trauma	1.5	0	1	0	0	0
**Cardiogenic**	**4.6**	**2**	**1**	0	0	0
**Miscellaneous**	**12.3**	**3**	**5**	**12.3**	**5**	**3**
Decubital ulcer	3.1	0	2	0	0	0
Torsion of abdominal organ	1.5	1	0	6.2	4	0
Spleen rupture	1.5	1	0	0	0	0
Rectal prolapse	1.5	0	1	0	0	0
Anaemia	1.5	0	1	1.5	0	1
Hypovolaemic shock, haemoabdomen	1.5	1	0	0	0	0
Gastric ulcer	1.5	0	1	1.5	0	1
Vaginal prolapse	0	0	0	1.5	0	1
Uterine prolapse	0	0	0	1.5	1	0
**Unknown, suspected cardiogenic**	**9.2**	**6**	**0**	**0**	**0**	**0**
**Unknown**	**13.8**	**4**	**5**	**0**	**0**	**0**
Unknown	12.3	3	5	0	0	0
Unknown due to putrefaction	1.5	1	0	0	0	0

PAD categories are written in boldface

One farm was overrepresented in the sample, providing 31% of all sows (*n* = 20). These animals represented the whole sample quite well, as 40% had an inflammatory and one-third a miscellaneous PAD-1, without any particularly prevalent diagnoses.

**Fig. 2:**
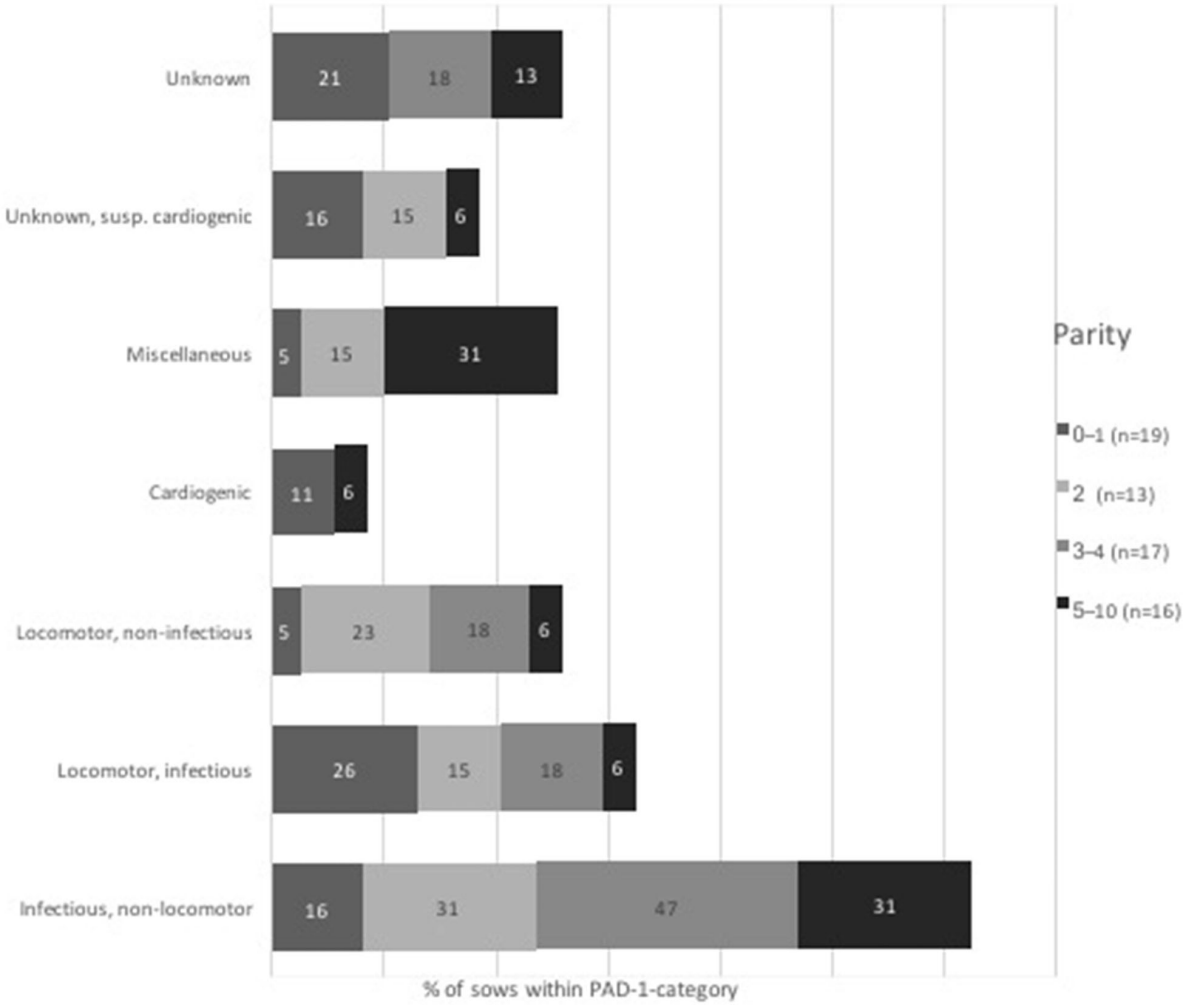
Parity distribution within categories of primary pathological-anatomical diagnosis (PAD-1) in a sample of dead and euthanized sows (*n* = 65) on 15 Finnish farms

An overview of the distribution of parity within the category of PAD-1 is shown in Fig. 2. Cardiogenic, inflammatory locomotor and unknown causes were relatively common in parity 0–1. Locomotor system PAD-1 s were important in young sows (parity 2–3), diminishing in older sows (parity 4–10), for which inflammatory diseases in other locations prevailed.

A PAD-2 was reported in 20 sows (31%) (Table 3). Incidental findings were present in all but one sow, and typically numerous (Table 4). The majority of sows presented with tooth wear (71%) and/or degenerative joint disease (63%). Some incidental findings, such as degenerative joint disease, tooth wear or mild pneumonia, were recorded in sows for which no PAD-1 could be determined.

**Table 4:** Incidental findings in post-mortem examination of 65 sows found dead (*n* = 27) or euthanized (*n* = 38) from 15 Finnish sow herds

	Found dead	Euthanized	% of all sows
Tooth wear	19	27	70.8
Degenerative joint disease	18	23	63.1
Skin lesions	9	20	44.6
Decubital ulcer			35.4
Grade 1–2^a^	8	10	
Grade 3–4^a^	1	4	
Periodontal disease	9	8	26.2
Pneumonia/pleuritis	2	15	26.2
Tooth fracture	7	9	24.6
Cystitis	3	11	21.5
Other joint disease	6	5	16.9
Miscellaneous	6	5	16.9
Tooth calculus	2	5	10.8
Gastric ulcer	2	4	9.2
Metritis	1	5	9.2
Nephritis	2	3	7.7
Hepatitis	0	4	6.2
Vulva lesion	0	5	6.2
Bruising	2	1	4.6
Enteritis	1	1	3.1
Arthritis	1	0	1.5
Bursitis	0	1	1.5
Gastritis	1	0	1.5
Mastitis	1	0	1.5
Myocarditis	0	1	1.5
Myositis	0	1	1.5
Pericarditis	0	1	1.5
Peritonitis	1	0	1.5

^a^Classified according to the shoulder with the highest grade. In grade 1, the ulceration is limited to epidermis; grade 4 is the most severe ulceration, where bone is exposed

Farmers categorized the cause of death correctly in 15%, partly correctly in 29% and incorrectly in 55% of cases.

### Post-mortem findings associated with the locomotor system

Post-mortem findings associated with the locomotor system were common in the present data, with 91% (*n* = 60) of the animals affected, most of which had more than one type of lesion or in more than one location. The locomotor PAD was primary in 19 (29%) and secondary in 6 sows (9%), whereas 63% (*n* = 41) presented with degenerative joint disease as an incidental finding.

**Fig. 3:**
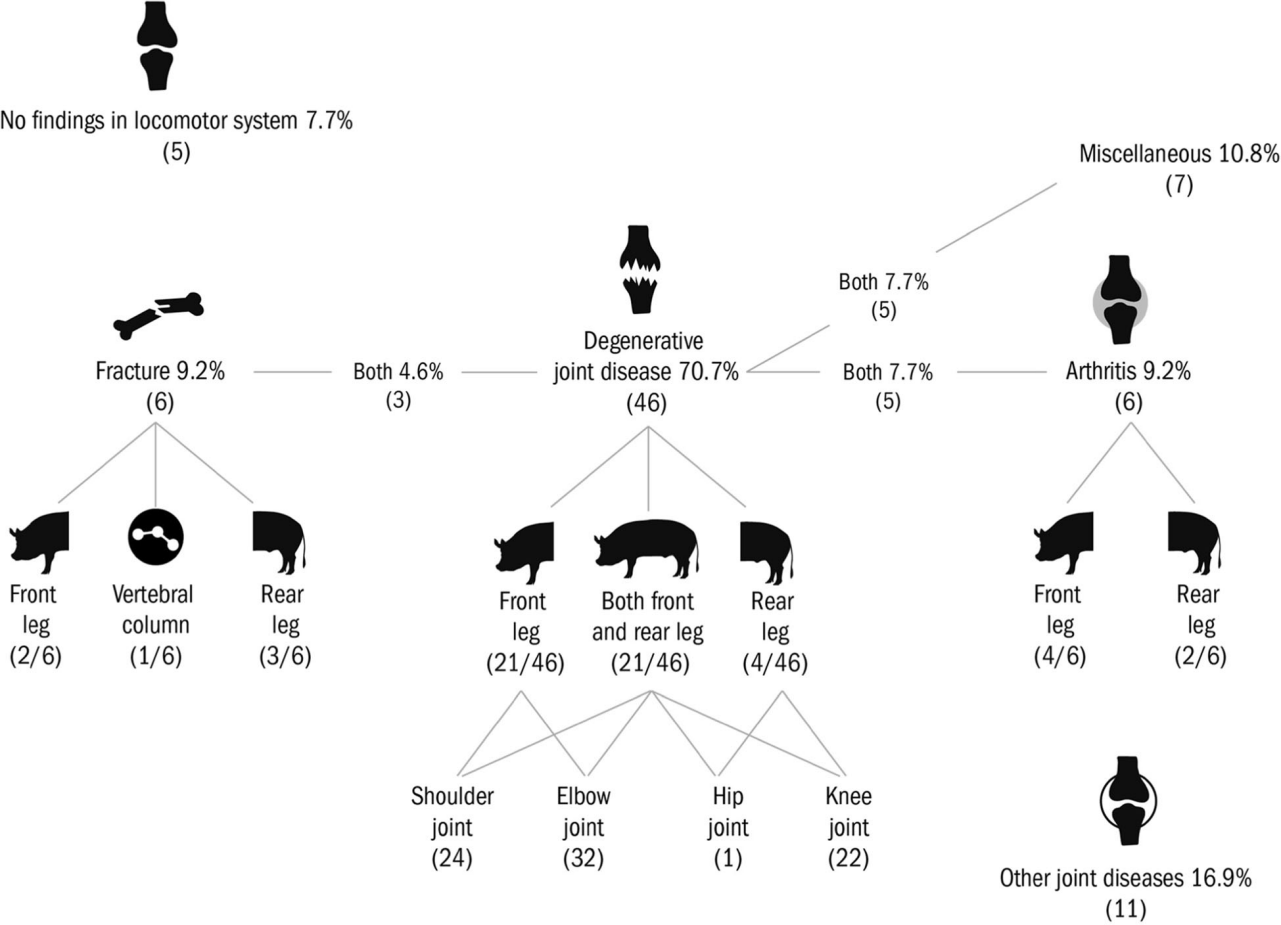
Findings associated with locomotor system in necropsy of 65 sows found dead or euthanized, given as a proportion (%) of all sows in the data, and the number of sows (in parentheses)

The type and distribution of locomotor findings are detailed in Fig. 3. Degenerative joint disease was diagnosed in altogether 46 animals (71%) (PAD-1, PAD-2 and incidental findings together). The elbow joint was most commonly affected (*n* = 32), followed by the shoulder (*n* = 24) and knee (*n* = 22). Degenerative joint disease was bilateral in two-thirds of the cases. Both front and rear leg were affected in 21 sows. The prevalence of degenerative joint disease was highest in young sows, with 50% of the cases occurring in parity 0–2 sows. Less common findings in the locomotor system included arthritis (*n* = 11), fractures (*n* = 8), other joint diseases (*n* = 11) and miscellaneous reasons (*n* = 12).

### Dental and periodontal disease

DPD was a frequent incidental finding, found in 74% of the animals. Young sows (parity 0–1) were numerically less affected by DPD than older ones (Table 5). Tooth wear was the most common DPD, with a prevalence of 71% (*n* = 46). Tooth fractures were present in 16 animals (25%), including five sows with one and 11 sows with more than one affected tooth. The frequency of tooth wear and fractures increased numerically with increasing parity. Signs of periodontal disease were recorded in 17 animals (26%), including gingival retraction (*n* = 5), gingivitis (*n* = 7), periodontitis (*n* = 1) and loose or missing teeth (*n* = 7). Dental calculus of at least moderate degree was present in 7 animals (11%). Infrequent DPD included superfluous incisors in the mandible suspected to be persisting primary teeth (*n* = 2) and caries (*n* = 1). Teeth change was in progress in one animal, and one had lesions on the tongue.

**Table 5:** Dental and periodontal lesions according to parity in 65 sows dead or euthanized on 15 Finnish farms. Prevalences are given as n with the finding (proportion within parity category). More than one finding per sow could be reported

Parity	0–1 (*n* = 19)	2 (*n* = 13)	3–4 (*n* = 17)	5–10 (*n* = 16)
Tooth wear	9 (47%)	10 (77%)	13 (77%)	14 (88%)
Fracture	3 (16%)	3 (23%)	4 (24%)	6 (38%)
Periodontal disease	5 (26%)	5 (39%)	3 (18%)	4 (25%)
Calculus	0	3 (23%)	2 (12%)	2 (13%)

## Discussion

### General comments

The majority of sows dead or euthanized on-farm were affected by locomotor and/or dental-periodontal disease. Multiple pathological findings were the norm. The generalizability of the results is questionable, given that the animals originated from a convenience sample of farms, with one farm heavily overrepresented. Moreover, the animals did not necessarily represent average OFD on the farms, as farmers could choose which sows to enrol in the study. Investigating the most frequent causes of death by performing numerous sow necropsies is of value in herds with a high mortality rate, but rarely provides useful information if they are done sporadically on the most complicated cases. Even if the results are considered merely descriptive, we believe that they contribute valuable data to this poorly known field.

### Causes of OFD

Inflammatory disease was the most common type of PAD-1 in this study, accounting for 48% of the animals. Of these, 11 were located in the locomotor system, and 20 elsewhere in the body. Our results indicate that disease in the reproductive organs seldom lead to OFD in this sample, as the reproductive system was involved in only two cases. The importance of this type of lesion as PAD-1 appears variable at least as judged by reports on 265 sows from 10 Danish herds [9] and 96 sows from a Swedish sow pool [10], where reproductive organ lesions comprised 14 and 3.1% of OFDs, respectively. Unfortunately, comparisons with previous results must be undertaken cautiously due to methodological differences or a lack of information on methodology.

The most prevalent PAD-1 s in the present study were arthritis and peritonitis, each accounting for 9% of the animals. Arthritis was reported to be the most prevalent cause of OFD in the Danish (prevalence 24%) [9], and Swedish (prevalence 36%) [10] data sets introduced above. Neither of these papers mention peritonitis as a cause for OFD, although it was one of the most prevalent PAD-1 s in our study, affecting 9.2% of the animals. However, Kirk et al. [9] found torsions of internal organs to be a common cause of OFD, affecting 21% of the sows. Given that three of altogether six peritonitis cases in our study appeared to be preceded by spleen or liver lobe torsion, we believe that the two studies are actually describing the same condition.

Decubital ulcers were the second most prevalent PAD in the sows, affecting 38% of the animals. DU was typically an incidental finding, and the cause for OFD in only two of the 25 animals. In both cases, the sow was euthanized. DU is considered a major welfare problem in the sow [16] and has been reported to increase the risk of mortality [7]. Known factors contributing to the development of DU include genetics, BCS and flooring, as reviewed by Rioja-Lang et al. [17]

Sanz et al. [18] reported gastric ulcer as a primary OFD cause in a sample of 107 necropsies from a large sow herd with high mortality. Gastric ulcers were infrequent in the present data. One reason for this discrepancy may be that several actions aiming to prevent gastric ulcers, including roughage provision and adequate grain particle size, are commonly undertaken on Finnish farms. Once-per-day feeding of sows, which was the major cause for the high prevalence of ulcers according to Santz et al. [18], was not practiced on the study farms.

### Pathological findings in the locomotor system

The locomotor system was the body part most commonly affected by lesion in this study, looking both at the PAD-1 s (29% of all animals) and incidental findings (85%). Nine of 10 sows were diagnosed with a locomotory PAD, with degenerative joint disease as the most common condition, present in 71% of the animals. Kirk et al. [9] reported an even higher prevalence of degenerative joint disease (i.e. arthrosis) in 265 OFD sows from 10 Danish herds, with 9 of 10 animals affected. In the animals here, degenerative joint disease was in many cases assumed to be secondary to osteochondrosis (OC). One factor suggestive of this aetiology was a bilateral presentation, which is typical for osteochondrotic lesions [19]. Osteochondrosis is caused by a focal disturbance in endochondral ossification, which is a result of failure of the blood supply to epiphyseal growth cartilage [20]. Pigs are commonly affected by OC. In the study of Etterlin et al. [21] all fattening pigs (*n* = 100) had OC on the talus when examined by computed tomography.

Although the study sample represents unhealthy animals selected by the farmer, the high frequency of locomotor system lesions as incidental findings raises questions about the situation of sows in intensive production in general. Lameness has been associated with decreased longevity [22] and reduced activity [23] in sows as well as with decreased feed intake in growing pigs [24]. Joint disorders are often progressive. In cattle, lameness is considered a chronic pain syndrome [25, 26]. Many of the sows here may have suffered from long-term pain. Pain originating in joints is known to include a neuropathic component, characterized by allodynia (pain in response to a normally innoculous stimulus) and hyperalgesia (increased pain intensity in response to a painful stimulus, reviewed by McDougall [27]).

### Dental and periodontal disease

Although none of the present primary pathological diagnoses involved the teeth or periodontium, findings in these areas were common and often multiple. Dental and periodontal disease receive special emphasis in this paper given their high prevalence, potentially significant effect on sow health and welfare and scarce availability of information in the scientific literature.

A study by Johnson et al. [28] showed presumably painful oral lesions in 85% of 82 commercial slaughtered sows in USA and Canada. Equally high prevalence of at least one type of DPD was found in the present animals (74%) and of tooth damage in 81 English cull sows (roughly 90%) by Davies et al. [29]. Engblom et al. [10] recorded tooth lesions, including missing, broken or severely worn teeth, in 96 OFD sows from a large Swedish farm and reported a prevalence of 31%.

Tooth wear was the most common DPD, with 71% of the present animals affected by at least moderate changes. These findings are in accordance with a few previous reports. Johnson et al. [28] described molar wear in 63% and incisor wear in 62% in 82 cull sows in USA and Canada, Davies et al. [29] reported wear in 90% of 82 English cull sows and Malmsten et al. [30] reported wear in 70% of 99 farmed wild boar in Sweden. Tooth wear appeared to be more prevalent with increasing age in the present sows. Johnson et al. [28] suggested, based on dental examination of 108 live sows, that molar wear was associated with age, whereas incisor wear was caused by some factor present in indoor, but not outdoor housing. This factor may be bar biting [29]. Advanced tooth wear may lead to pulpitis and subsequent periodontitis, and severe wear in man is associated with oral pain and dysfunction [31]. Johnson et al. [28] also reported pain at probing of worn teeth in live sows.

The second most prevalent dental finding in the present sows was periodontal disease, affecting 26% of the animals. Previous reports give similar or higher prevalences. Roughly every fourth feral and domestic pig in an Australian sample of 159 sows was affected [32]. Malmsten et al. [30] reported tooth loss in 47% and severe periodontitis in 16% of 99 wild boars in Sweden, whereas slaughter sows had significant tartar and/or gingivitis, incisor loss or abscessation of periodontal pockets in 55, 34 and 4% of cases, respectively [28].

One-fourth of the present sows had at least one fractured tooth. We believe that fractures are initiated as trauma due to chewing on or colliding with hard objects such as fixtures. Fractures have been reported in semi-natural conditions as well, with a prevalence of 8% in wild boars [30]. Johnson et al. [28] noted that broken teeth in sows were painful and that affected sows in two study herds lost weight and were culled. Periodontal disease is a well-known risk factor for several systemic conditions in man, as reviewed by Igari et al. [33], and could thus have contributed to some of the conditions diagnosed in the present sows. We did not attempt to investigate associations between DPD and BCS, or other pathologies, due to the multitude of findings in the animals.

### Multitude of pathological findings

Pathological findings are expected in sows that die on-farm. The large number of different pathologies was, however, surprising. Multiple PADs were the norm, with all but one of the sows presenting with two or more PAD types. In one-third (31%) of the animals, a PAD-2 was identified as possibly contributing to death in addition to the PAD-1. The literature provides very little information to put these findings into perspective. Moreover, lack of detail in descriptions of the methods complicates comparisons. Sanz et al. [18] reported that 57% of 107 sows from one herd had at least two types of lesions. Engblom et al. [10] listed numerous incidental findings, but did not report the proportion of sows having several pathologies. In a study evaluating gross lesions of over 3000 cull sows from harvest plants, the coexistence of multiple lesions was common [34].

Multiple pathologies in an animal may indicate that the course of illness was prolonged and medical treatment and/or euthanasia thereby delayed. This obviously raises concerns about the welfare of the sample sows, and perhaps also sows in commercial piglet production in general. The study design, allowing the farmer to select which sows to sign up, may, however, have led to an overrepresentation of complicated or less obvious cases.

### Differences according to the means of death and effects of age

Although the study sows were not random samples of OFD on the farms, a few apparently significant effects of the means of death and parity will be discussed below. These include BCS, timing of death in relation to production phase and the distribution of certain PME findings.

Body weight or parity was not associated with the means of death, whereas euthanized sows had significantly lower BCS than sows found dead (average BCS 2.5 vs. 2.9). Kirk et al. [9] reported a similar effect in OFD sows from 10 Danish herds. This relationship may be at least partly explained by the timing of death. Cases of euthanasia were clustered to the immediate post-weaning period, when sows are usually at their thinnest, as opposed to the more uniform distribution across production phases of spontaneous deaths. This practice probably reflects the farmers’ desire to bring the litter to the weaning stage despite sickness in the sow, which may cause prolonged suffering for the sow. A similar clustering of euthanasia was described in two Swedish data sets that included one farm [10] and 21 farms [1].

Spontaneous deaths were in this data distributed according to the length of the production phases, except for a numerical clustering immediately pre-farrowing. Of 19 sows dying between the 16.5-week period between insemination and farrowing, four took place in the short period (days to a week) spent in the farrowing pen pre-farrowing. This effect may reflect the high level of stress in the animals at this time, comprising both physical and metabolic stress due to late-stage pregnancy, but most probably also psychological stress from being confined in a crate after loose housing. In a Swedish study on removal patterns on 21 farms, spontaneous deaths were clustered post-farrowing [1]. Sasaki and Koketsu [35] reported an increased risk of OFD (both spontaneous death and euthanasia) in the peripartum period on 105 Japanese farms. Our results cannot, however, be effectively compared with these due to differences in study design.

Certain PAD patterns could be observed according to means of death and parity. There was a more uniform distribution of different pathologies in spontaneously dead sows than in euthanized sows. A PAD-1 or PAD-2 affecting the locomotor system was significantly more frequent in euthanized sows than in spontaneously dead sows (24 of 38 vs. 1 of 27 sows), which is in accordance with data from one Swedish farm [10].

Spontaneous deaths took place mainly due to unknown, suspected cardiogenic causes (6 of 27 sows) and peritonitis (5 of 27 sows). In the data by Engblom et al. [10], cardiogenic causes and miscellaneous trauma were the most important causes, both accounting for 4 cases of 17. In contrast, Kirk et al. [9] reported lesions in gastrointestinal or reproductive systems as the main causes of spontaneous death.

Most cases of euthanasia were due to infections, which were present in a large number of different locations in the study animals. The most prevalent PAD-1 s were arthritis (6 of 38 sows) and abscess (3 of 38 sows).

Inflammatory locomotor causes of death were frequent in young sows (parity 0–1). These animals may have been unsuitable for the production environment due to, for instance, osteochondrosis, which is a prevalent condition in young pigs [21]. Pigs affected by any primary orthopaedic condition causing pain and/or clumsiness may have an increased risk for bruising of the skin with introduction of infectious agents, if housed without proper bedding.

Locomotor lesions in general clearly peaked as a PAD-1 in parity 2–3 sows, decreasing substantially thereafter. Although most sows had some locomotor system lesion, it was infrequent as the primary cause of death from parity 4 onwards. Parity 2–3 may be the time that a locomotor system with a suboptimal anatomy or some other congenital weakness typically tolerates the production environment. Inflammatory diseases were frequent causes of death in old sows, perhaps due to accumulation of risks, including births and number of contacts with other animals.

### Pre-mortem signs and medications

The rate of animals dying spontaneously is considered a potential sign of decreased welfare on a farm [4], given that at least some of these deaths will be in animals that should have been euthanized due to severe sickness. The proportion of sows found dead without any signs of disease reported was 52% (14 of 27 sows). We believe that these animals had showed some signs of disease, which were not recognized by the caretakers in many cases. A similar result was reported by Sanz et al. [18] on one farm, where sickness had been detected in only half of spontaneously dead sows. In the present data, the farmer’s impression of the cause of death was at least partly correct in 44% of the cases, which agrees with previous data from dairy farms [11].

Lameness was the most commonly observed sign preceding euthanasia in the animals here, consistent with Kirk et al. [9] and Engblom et al. [10], which is as expected given the importance of locomotor lesions in both this and the cited studies. Lameness is relatively easily identified by farmers [36]. Still, it often remains unclear whether it is identified accurately. The farmers of this study reported lameness in the leg with the most severe finding in half of the 30 cases where lameness or inability to stand up were reported. The rather low figure may be explained by the high prevalence of lesions in more than one leg, which could have complicated allocation of the sign. Locomotive characteristics are also known to correlate poorly with joint lesions, at least in the case of macroscopic osteochondrosis lesions in growing gilts [37].

Of the animals dying spontaneously after showing signs of sickness, 10 of 13 had been medicated. Attempts had thus been made to resolve the situation. The proportions of medicated animals according to means of death were in line with the results reported by Engblom et al. [10]. They noted that 58 and 29% of euthanized and spontaneously dead sows, respectively, had been medicated, compared with the 63 and 37% in our study.

## Conclusions

The very high prevalences of pathological processes found in this convenience sample of sows dying or euthanized on-farm raises concerns about the health status and welfare of sows in intensive piglet production in general. Degenerative joint disease and dental-periodontal disease were present as incidental findings in the majority of animals, indicating an urgent need to establish the prevalence and clinical relevance of these disorders in the general sow population. The different causes of unexpected sow death should be thoroughly investigated on a farm-by-farm basis to guide the necessary control measures in order to decrease mortality and avoid unnecessary delay in euthanasia of unhealthy sows.

## Supplementary information

**Additional file 1.** A questionnaire to collect general information on the sow and on the signs and circumstances preceding death or euthanasia.

**Additional file 2.** The standard operation procedure for post-mortem examination.

## Data Availability

The data analysed during this study are available from the corresponding author upon reasonable request.
