# Supplementary material for: Pathological findings in spontaneously dead and euthanized sows – a descriptive study
Source: Porcine Health Manag. 2019 Nov 20;5:25. doi: 10.1186/s40813-019-0132-y (PMC6864960; doi:10.1186/s40813-019-0132-y)
Supplement: Supplementary file 1 — Additional file 1. A questionnaire to collect general information on the sow and on the signs and circumstances preceding death or euthanasia. [file 40813_2019_132_MOESM1_ESM.docx]

| **Please fill in clearly** | | | | | **^DATE Dnro^** | | | | | | | | | | | | | |
| --- | --- | --- | --- | --- | --- | --- | --- | --- | --- | --- | --- | --- | --- | --- | --- | --- | --- | --- |
| ^Name^ | | | | | ^Address^ | | | | | | | | | ^Telephone^ | | | | |
| ^Herd number^ | | | | | ^Building number^ | | | | | | | | |  |  |  |  |  |
| ^Send the^  Answer and bill to : | | | | | SOW LONGEVITY PROJECT  University of Helsinki  Paroninkuja 20  04920 Saarentaus  mari.heinonen@helsinki.fi | | | | | | | | | +358-50-3182310 | | | | |
| send the answer to the owner also | | | | | | | | | | | | | | | | | | |
|  | | | | | | | | | | |  | | | | | | | |
| Sow ID number (ear tag number) | | | | |  | | | | | | Other ID | | | |  | | | |
| Date of birth ___ / ___ 201___ | | | | | | | | | | | | | | | | | | |
| Date of death and which day of the week it was | | | | | ___ / ___ 201___ day of the week _______________ | | | | | | | | | | | | | |
| Death or euthanasia | | | | |  | | 59 DIED | | | | | | | | | | | |
|  |  |  |  |  |  | | 29 EUTHANASIA | | | | | METHOD TO EUTHANISE  bolt gun and bleeding  shooting  with medicines | | | | | | |
| THE LATEST HOUSING PLACE FOR THE SOW | | | | |  | | LOOSE HOUSING | | | | | | | | | | | |
|  |  |  |  |  |  | | GROUP PEN, HOW MANY SOWS IN THE GROUP:  **______** | | | | | | | | | | | |
|  |  |  |  |  |  | | CAGE IN BREEDING UNIT | | | | | | | | | | | |
|  |  |  |  |  |  | | FARROWING PEN | | | | | | | | | | | |
| TEMPERATURE OF THE ROOM HOUSING THE SOW LATEST | | | | | | | | | | | | | | | | ° C | | |
| HOW WAS THE CARCASS HANDLED IN THE PIGGERY (describe with words) | | | | | | |  | | | | | | | | | | | |
| HOW WAS TEH SOW STORED IN THE PIGGERY BEFORE SENDING TO AUTOPSY, INFORM ALSO THE TEMPERATURE OF THE ROOM (describe with words) | | | | | | |  | | | | | | | | | | | |
|  | | | | | | | |  |  | | | | | | | | | |
| THE LATEST PARITY: ___________ THE DATE OF THE LATEST FARROWING ___ / ___ 20__ | | | | | | | | | | | | | | | | | | |
| THE LAST DATES FOR THESE OCCASIONS IN THE LIFE OF THE SOW | | | | | |  | BREEDING | | | | | | ___ / ___ 20__ | | | | | |
|  |  |  |  |  |  |  | FARROWING | | | | | | ___ / ___ 20__ | | | | | |
|  |  |  |  |  |  |  | WEANING | | | | | | ___ / ___ 20__ | | | | | |
|  | | | | | |  |  | | | | | | | | | | | |
| MEDICATIONS AND TREATMENTS BEFORE THE DEATH OF THE SOW (*Name and dose of the medicine, how many days did she receive the medication). DESCRIBE OTHER MEASURES DONE FOR THE SOW* | | | | | | | | | | | | | | | | | | |
|  | | | | | | | | | | | | | | | | | | |
| SIGNS OF DISEASE BEFORE DEATH (you can select more than one symptom) | | | | | | | | | | | | | | HOW MANY DAYS | | | | |
|  | 50 No signs before death | | | | | | | | | | | | |  | | | | |
|  | 01 Abortion | | | | | | | | | | | | |  | | | | |
|  | 02 Aggressive sow | | | | | | | | | | | | |  | | | | |
|  | 23 Respiratory signs | | | | | | | | | | | | |  | | | | |
|  | 24 Changes in the skin or hair | | | | | | | | | | | | |  | | | | |
|  | 25 Lameness | | Right frontleg  Left frontleg  Right hindleg  Left hindleg | | | | | | | | | | |  | | | | |
|  |  |  |  |  |  |  |  |  |  |  |  |  |  |  |  |  |  |  |
|  |  |  |  |  |  |  |  |  |  |  |  |  |  |  |  |  |  |  |
|  |  |  |  |  |  |  |  |  |  |  |  |  |  |  |  |  |  |  |
|  | 28 Fever | | | | | | | | | | | | |  | | | | |
|  | 33 Nervous signs | | | | | | | | | | | | |  | | | | |
|  | 34 Diarrhea | | | | | | | | | | | | |  | | | | |
|  | 37 Digestive system, other signs than diarrhea | | | | | | | | | | | | |  | | | | |
|  | 38 Anorexia | | | | | | | | | | | | |  | | | | |
|  | 39 Repeat breeding | | | | | | | | | | | | |  | | | | |
|  | 40 Vomiting | | | | | | | | | | | | |  | | | | |
|  | 41 Changes in urine | | | | | | | | | | | | |  | | | | |
|  | 47 Behavioural changes | | | | | | | | | | | | |  | | | | |
|  | 51 Paralysis / severe problem in locomotory system / not able to stand up | | | | | | | | | | | | |  | | | | |
|  | 52 Vaginal discharge | | | | | | | | | | | | |  | | | | |
|  | Agalactia | | | | | | | | | | | | |  | | | | |
|  | Mastitis | | | | | | | | | | | | |  | | | | |
|  | Abscesses | | | | | | | | | | | | |  | | | | |
|  | Farrowing problems | | | | | | | | | | | | |  | | | | |
|  | Accident | | | | | | | | | | | | |  | | | | |
|  | Other, explain, | | | | | | | | | | | | |  | | | | |
|  | | | | | | | | | | | | | | | | | | |
| THE MOST LIKELY REASON OF DEATH IN THE OPINION OF THE OWNER | | | | | | | | | | | | | | | | | | |
|  | | | | | | | | | | | | | | | | | | |
| DO OTHER SOWS IN THE HERD HAVE SIMILAR SIGNS OF DISEASE | | | | YES  NUMBER OF SOWS WITH SIGNS ___________ | | | | | | | | | | | | | | NO |
|  | | | | | | | |  | |  | | | | | | |  | |
| ANY OTHER INFORMATION | | | | | | | | | | | | | | | | | | |
| This information can be attached to the autopsy results. | | | | | | | | | | | | | | | | | | |
| ^Date^  ___ / ___ 201____ | | ^Signature^ | | | | | | | | | | | | | | | | |
