# Supplementary material for: Pathological findings in spontaneously dead and euthanized sows – a descriptive study
Source: Porcine Health Manag. 2019 Nov 20;5:25. doi: 10.1186/s40813-019-0132-y (PMC6864960; doi:10.1186/s40813-019-0132-y)

**Evira/TUVI/Production animals 2014/** Sow longevity –samples (University of Helsinki)

**SOW OBDUCTION – FINDINGS**

**Basic information:**

Number:__________________ PAT DNRO:____________ Sow ID number:____________ Weight:_________ (□estimated), girth measure ______cm and from head to tail_____ cm

Age:_________________ □ Euthanised □ Died

**1. Body condition and skin**

**Body condition**_____________________________________________________________

**Skin:** □ No changes □ Changes: ______________________________________________________

______________________________________________________________________________________

**Shoulder are, skin etc. :** □ No changes □ Changes (see below 1-4): Right:_______ Left:_________

(0= No changes, 1= Changes in epidermis, superficial scar, 2= Changes also in dermis, some scar tissue, 3= Changes reach subcutaneous tissue, remarkable scar tissue, 4= tuber spina scapulae can be seen or felt easily

**2. Joints and claws:**

**Shoulder joint:**  □ No changes □ Changes: ______________________________________________

**Elbow joint:** □ No changes □ Changes: ______________________________________________

**Carpus:** □ No changes □ Changes: _______________________________________________

**Hip joint:** □ No changes □ Changes: _______________________________________________

**Knee joints:** □ No changes □ Changes: _______________________________________________

**Hock joints:** □ No changes □ Changes: _______________________________________________

**Claws** (Symmetry, length, fissures): ______________________________________________________

______________________________________________________________________________________

**3. Oral cavity:** □ No changes □ Changes:________ ________________________________________

**Teeth:** □ No changes □ Changes (See below)

Upper Jaw (I3/3, C1/1, P4/4, M3/3)

DX SIN

| M3 | M2 | M1 | P4 | P3 | P2 | P1 | C | I3 | I2 | I1 | I1 | I2 | I3 | C | P1 | P2 | P3 | P4 | M1 | M2 | M3 |
| --- | --- | --- | --- | --- | --- | --- | --- | --- | --- | --- | --- | --- | --- | --- | --- | --- | --- | --- | --- | --- | --- |
|  |  |  |  |  |  |  |  |  |  |  |  |  |  |  |  |  |  |  |  |  |  |

Changes, which:__________________________________________________________________________

Lower Jaw (I3/3, C1/1, P4/4, M3/3)

DX SIN

| M3 | M2 | M1 | P4 | P3 | P2 | P1 | C | I3 | I2 | I1 | I1 | I2 | I3 | C | P1 | P2 | P3 | P4 | M1 | M2 | M3 |
| --- | --- | --- | --- | --- | --- | --- | --- | --- | --- | --- | --- | --- | --- | --- | --- | --- | --- | --- | --- | --- | --- |
|  |  |  |  |  |  |  |  |  |  |  |  |  |  |  |  |  |  |  |  |  |  |

Changes, which:__________________________________________________________________________

**4. Chest cavity:**

**Liquid** (amount and quality): _____________________________________________________________

**Trachea: □** No changes □ Changes Contents:____________________________________________________


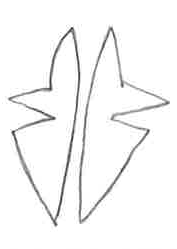


**Lungs:**

□ No changes

□ Changes:_______________________________________________

__________________________________________________________

__________________________________________________________

__________________________________________________________


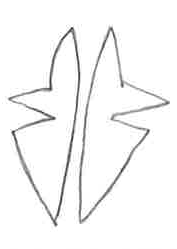


**Pleura:**

□ No changes

□ Changes:________________________________________________

___________________________________________________________

**Heart:**

□ No changes □ Changes:________________________________

___________________________________________________________

**5. Abdominal cavity:**

**Liquid contents** (amount and quality): _______________________________________________________________ **Position of internal organs, peritoneum:**  □ No changes

□ Changes: ___________________________________________________________________________

______________________________________________________________________________________

**Liver:** □ No changes □ Changes:_______________________________________________

**Spleen:** □ No changes □ Changes:_______________________________________________

**Intestinal tract:**

**Stomach:** □ No changes □ Changes:______________________________________

**Small intestine:** □ No changes □ Changes: _______________________________________________

**Large intestine:** □ No changes □ Changes: _______________________________________________

**Rectum:** □ No changes □ Changes: _______________________________________________

**Lymph nodes in intestinal trackt:**  □ No changes □ Changes:_________________________________

**Urinary tract:**

**Kidneys:** □ No changes □ Changes: _______________________________________________

**Urinary bladder:** □ No changes □ Changes: _______________________________________________

______________________________________________________________________________________________________________________________________________________________________________

**Reproductive organs:**

**Uterus:** □ No changes □ Changes: _____________________________________________________

**Ovaries:** □ No changes □ Changes: _________________________________________________

**Lymph nodes in pelvic area** □ No changes □ Changes: _________________________________

______________________________________________________________________________________________________________________________________________________________________________

**Samples taken in connection with the field obduction, and other notes:**

Histology □ lungs □ heart □ liver □ spleen □ kidney □ bladder

□ tissues with changes, which: __________________________________________________

Bacteriology □ lungs □ heart □ liver □ spleen □ kidney □ bladder

□ tissues with changes, which: __________________________________________________ □ samples taken into transport media, tell which: ____________________________________________________________________


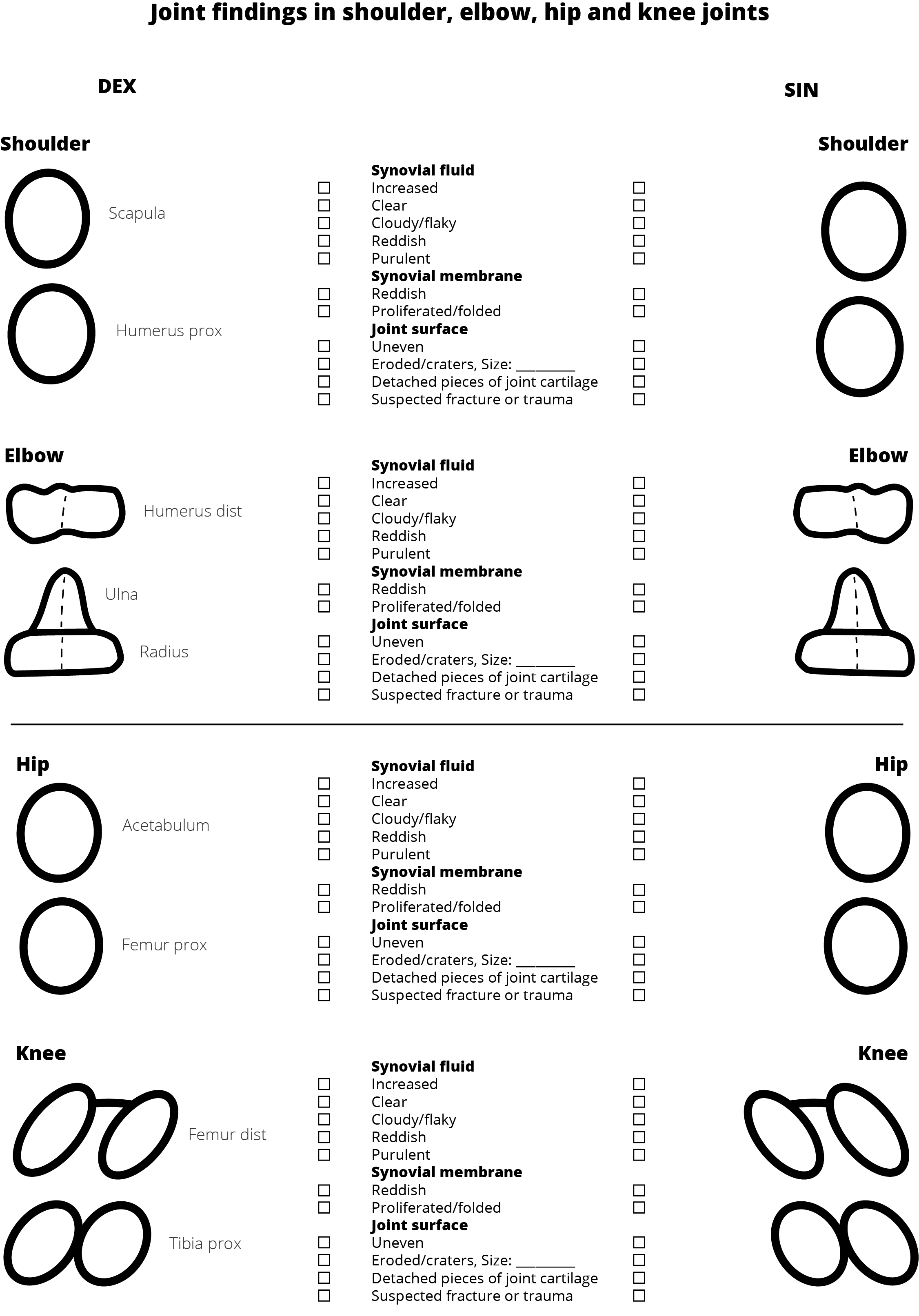

Supplement: Supplementary file 2 — Additional file 2. The standard operation procedure for post-mortem examination. [file 40813_2019_132_MOESM2_ESM.docx]
